# Does early introduction of solid feeding lead to early cessation of breastfeeding?

**DOI:** 10.1111/mcn.12944

**Published:** 2020-01-29

**Authors:** Angelina Lessa, Ada L. Garcia, Pauline Emmett, Sarah Crozier, Sian Robinson, Keith M. Godfrey, Charlotte M. Wright

**Affiliations:** ^1^ Department of Human Nutrition School of Medicine, Nursing and Dentistry University of Glasgow Glasgow UK; ^2^ Centre for Child and Adolescent Health, Population Health Sciences, Bristol Medical School University of Bristol Bristol UK; ^3^ MRC Lifecourse Epidemiology Unit University of Southampton Southampton UK; ^4^ NIHR Southampton Biomedical Research Centre University of Southampton and University Hospital Southampton NHS Foundation Trust Southampton UK; ^5^ Department of Child Health, School of Medicine, Nursing and Dentistry University of Glasgow Glasgow UK; ^6^ NIHR Newcastle Biomedical Research Centre Newcastle upon Tyne Hospitals NHS Foundation Trust and Newcastle University Newcastle upon Tyne UK

**Keywords:** ALSPAC, breastfeeding, cohort study, complementary feeding, infant, lactation

## Abstract

Mixed milk feeding increases the likelihood of breastfeeding cessation, but it is not known if solid feeding (SF) has the same effect. We have identified 10,407 infants breastfed for at least 8–10 weeks from three large U.K. studies (Avon Longitudinal Study of Parents and Children [ALSPAC; born 1990–1991], Southampton Woman's Survey [SWS; 1998–2008], and Infant Feeding Survey 2010 [IFS 2010]) to investigate the associations between early SF and breastfeeding cessation. In the earliest study (ALSPAC), 67% had started SF before the age of 4 months, but in the latest (IFS), only 23% had started before 4 months. Solid food introduction before 4 months was associated with stopping breastfeeding before 6 months in all three cohorts, with little effect of adjustment for maternal sociodemographic characteristics (Poisson regression, adjusted prevalence ratios: ALSPAC 1.55, [95% confidence interval 1.4, 1.8], SWS 1.13 [1.0, 1.3], IFS 1.10 [1.1, 1.3]). Using Cox regression, adjusted hazard ratios for breastfeeding cessation compared with SF after 5 months were 2.07 (1.8, 2.4) for SF before 4 and 1.51 (1.3, 1.8) at 4–5 months for ALSPAC and 1.25 (1.1, 1.5) and 1.15 (1.0, 1.3) for SWS. Earlier introduction of solids was associated with a shorter duration of breastfeeding, particularly in cohorts where earlier introduction of solids was the norm, with a dose–response relationship, which was not explained by background social characteristics. As mothers most commonly introduced solids in the month prior to the then recommended age, continuing to recommend deferring solids to the age of 6 months is important to support sustained breastfeeding.

**Key messages**

- It is well recognised that intake of breast milk is displaced by use of infant formula milk and that this is an important cause of early breastfeeding cessation, but it is less clear whether early introduction of solid foods has the same effect.
- This paper studied breastfeeding mothers in three large representative U.K. cohorts completed in the last 25 years and found that mothers most commonly introduced solids in the month prior to the recommended age at that time.
- In all three cohorts, introduction of solid foods before 5 months was associated with a shorter duration of breastfeeding, and those starting solid foods before 4 months were more likely to stop breastfeeding prematurely.

## 1 INTRODUCTION

Breastfeeding is important throughout the first year of life, with positive health outcomes in the short (Horta & Victora, 2013) and long term (Kramer et al., 2008). These protective effects are most pronounced in low‐ and middle‐income countries but are also important in high‐income countries (Victora et al., 2016). For this reason, in 2002, the World Health Organisation recommended breastfeeding exclusively for 6 months, followed by continued breastfeeding with complementary feeding up to 2 years or beyond (Kramer & Kakuma, 2002). This position was endorsed by the U.K. Department of Health in 2003 (Health., 2003), thus deferring the recommended onset of solid feeding (SF) by 2 months from the previous recommendation of 4–6 months. Early on, it was suggested that this later introduction of SF could be harmful (Fewtrell, Wilson, Booth, & Lucas, 2010), but a recent detailed review has found no evidence for this (SACN, 2018). Most recently, a draft European review has concluded that there is no nutritional benefit from starting solids before 6 months (EFSA, 2019). Equally, the review suggested that there was no evidence of harm in developed countries, but did not consider the possible impact of early SF on exposure to breast milk.

The use of formula milk has long been known to impact on the duration of breastfeeding (Hornell, Hofvander, & Kylberg, 2001; Maijaliisa Erkkola et al., 2009). The early introduction of solid foods into the infant's diet will lead to some displacement of breast milk (Wells et al., 2012) and thus may increase the likelihood of early termination of breastfeeding (Noble & Emmett, 2006). However, systematic reviews addressing the influence of other foods and drinks on breastfeeding patterns have not identified enough evidence to draw conclusions about a link between exposure to SF and breastfeeding duration (Becker & Remmington, 2014; Szajewska, Horvath, Koletzko, & Kalisz, 2006). Our aim therefore was to undertake a secondary analysis of three large population‐based datasets to explore the extent to which starting solids early predicts shorter breastfeeding duration.

## 2 METHODS

This was a secondary analysis using three large U.K. longitudinal and population‐based studies, from which we identified children who were still receiving breast milk at an age when the introduction of solids could potentially interfere with continued breastfeeding.

The Avon Longitudinal Study of Parents and Children (ALSPAC) studied infants born between 1991 and 1992 (Boyd et al., 2013; Fraser et al., 2013); the Southampton Woman's Survey (SWS) studied infants born between 1998 and 2007 (Inskip et al., 2006), and the Infant Feeding Survey 2010 (IFS) was a U.K.‐wide survey conducted with mothers who gave birth in 2010 (McAndrew et al., 2012).

Table 1 compares the main characteristics of the three studies. The ALSPAC and SWS recruited women who lived in two specific geographical areas in Southern England before they gave birth. In ALSPAC, pregnant women resident in Avon, UK, with expected dates of delivery from April 1, 1991, to December 31, 1992, were invited to take part in the study. The number of pregnancies enrolled was 14,541 (at least one questionnaire returned or a clinic attended by 19/07/99). Of these pregnancies, there were 14,062 live births, and 13,988 children were alive at 1 year of age. Mothers provided information about themselves and their children via postal questionnaires at 4 weeks and 6 and 15 months after giving birth. 1Please note that the study website contains details of all the data that are available through a fully searchable data dictionary and variable search tool and reference the following webpage: http://www.bristol.ac.uk/alspac/researchers/our-data/



**Table 1:** Main characteristics of the three studies

	ALSPAC	SWS	IFS 2010
Years of birth of infants	1991–1992	1998–2008	2010–2011
Location	South west of England	Southampton	Across United Kingdom
Number of participants	13,988	3,158	10,768 (second stage)
Number of participants with breastfeeding information	10,256	2,873	7,875
Number (%) who ever breastfed	7,669 (75)	2,347 (82)	5,676 (72)
Number (%) of cohort still breastfeeding at the age of 8 weeks (10 weeks for IFS 2010)	6,079 (59)	1,500 (52)	2,828 (36)
Recruitment	In pregnancy	Prior to pregnancy	After birth
Age of first solid feeding	*N* = 6,031	*N* = 1,467	*N* = 2,749
<4 months	4,092 (68%)	289 (20%)	577 (21%)
4 months	1,641 (27%)	807 (55%)	875 (32%)
≥5 months	298 (5%)	371 (25%)	1,297 (47%)
Breastfeeding duration	*N* = 6,079	*N* = 1,500	*N* = 2,828
Two to three completed months^a^	1,525 (25%)	351 (23%)	775 (27%)
Four to five completed months	960 (16%)	370 (25%)	464 (16%)
Six or more months	3,594 (59%)	779 (52%)	1,589 (56%)

a In IFS, bands are 10 weeks to 4 months, >4 to 5 months, and > 5 months.

Abbreviations: ALSPAC, Avon Longitudinal Study of Parents and Children; SWS, Southampton Womans Survey; IFS 2010, Infant Feeding Survey 2010.

In the SWS, women were recruited before pregnancy, and for those who subsequently became pregnant, the resulting children were surveyed at ages 6 months and 1 and 2 years during home visits.

The IFS survey took a representative sample of mothers from all four countries of the United Kingdom, with births recorded during August and October 2010. This survey collected data through questionnaires sent to the mothers at infant ages 6, 26, and 38 weeks. In the IFS, many mothers dropped out before the 8‐month survey point, and not all of these had complete breastfeeding information, so the sample included were only those with data from the 8‐month survey with complete breastfeeding information (McAndrew et al., 2012).

### 2.1 Measures

Although each study used different questionnaires and survey techniques, for this analysis it was possible to extract comparable infant feeding variables from each dataset, albeit with some minor differences. For the present analysis we included children who were breastfed for at least 8 weeks. In the SWS and ALSPAC cohorts, duration of any breastfeeding was recorded in completed calendar months, so that children still breastfeeding after two completed months were included. In the IFS, breastfeeding duration was recorded only in discrete age categories (10 weeks or less, more than 10 weeks and up to 4 months, more than 4 months and up to 5 months, and so on), so infants still breastfeeding at 10 weeks were included. Duration of breastfeeding was recorded till the age of 24 months for SWS, 15 months for ALSPAC, and 9 months for IFS. For all three cohorts, the principle outcome of continued breastfeeding at 6 months was used. For SWS and ALSPAC, this was defined as 6 months and above, whereas for IFS, it was defined as more than 5 months and up to 6 months, or longer. For ALSPAC and SWS, the duration of any breastfeeding was also considered.

The main independent variable for all data sets was the age of SF introduction recorded in weeks. Three possible confounders, which are well‐known predictors of both breastfeeding duration and solids feeding, were available; two of these, mother's age and social class, were in all three datasets, whereas educational level was available for ALSPAC and SWS, although grouped slightly differently. Social class was collected according to the woman's original employment (Office of Population Censuses Surveys 1990), with six ranked groupings used in ALSPAC and SWS but only four for IFS (Table 2).

**Table 2:** Number (%) of participants who had been breastfeeding at the age of 8–10 weeks who were still breastfeeding at or beyond 6 months, by age of first solids feeding, maternal age, education, and social class

Predictor	ALSPAC	*p* d	SWS	*p* d	IFS	*p* d
	Number (%)		Number (%)		Number (%)	
Overall	3,594 (59.1)		779 (51.9)		1,589 (56.2)^e^	
Solid food introduced						
<4 months	2,218 (54.2)	*<.*001	130 (45.0)	.019	297 (51.5)	.008
4 months	1,120 (68.3)		419 (51.9)		491 (56.1)	
≥5 months	230 (77.2)		202 (54.4)		755 (58.2)	
Mother's age						
24 years or less	313 (43.4)	*<.*001	24 (32.0)	.003	130 (53.3)	.630
25 to 34 years	2,707 (59.7)		619 (52.3)		1,036 (56.5)	
35 years or more	574 (69.5)		136 (56.2)		420 (56.5)	
Mother's highest educational qualifications						
None/CSE^a^	297 (48.4)	.0001	40 (38.5)	<.001	‐	
Vocational training^a^	191 (47.3)		‐			
O level^a^	1,001 (51.4)		137 (40.8)			
A level^b^	1,093 (37.4)		223 (49.6)			
HND^c^	‐		68 (53.5)			
University degree	953 (76.2)		308 (64.2)			
Social class–maternal						
Professional	363 (78.1)	<.001	64 (66.7)	.003		
Management/technical	1,280 (63.8)		336 (54.5)		‐	
Skilled nonmanual	1,038 (52.4)		229 (46.9)			
Skilled manual	159 (56.0)		51 (49.5)			
Partly skilled	206 (55.5)		72 (50.0)			
Unskilled	32 (57.1)		7 (36.8)			
Social class–maternal						
Professional/managerial					902 (58.6)	.0011
Intermediate occupations	‐		‐		272 (54.3)	
Routine/manual occupations					249 (51.7)	
Never worked					44 (46.8)	

a Completed by the age of 16.

b Completed at the age of 18.

c Equivalent to 2 years at university.

d Chi^2^ linear trend.

e In ALSPAC and SWS, this was the number still feeding at or beyond six completed months. For IFS, this was the number still feeding beyond 5 months and up to 6 months, or beyond.

Abbreviations: ALSPAC, Avon Longitudinal Study of Parents and Children; SWS, Southampton Womans Survey; IFS 2010, Infant Feeding Survey 2010; CSE, Certificate of Secondary Education; HND, Higher National Diploma.

### 2.2 Analysis

All statistical analyses were performed using IBM SPSS Statistics 22.0 (IBM corp., 2013).

For all analyses, the predictor variable of age of solid food introduction was grouped into three categories: less than 4 months, four to less than five, and five calendar months or more.

To evaluate the effect of solid food introduction on breastfeeding cessation by 6 months, Poisson regression with robust variance was used to calculate the prevalence ratio for breastfeeding cessation before 6 months in all three cohorts and the crude and adjusted prevalence ratio obtained from a multivariable analysis.

In addition, in the ALSPAC and SWS datasets, survival analysis of the breastfeeding duration variable was undertaken. A new variable to indicate breastfeeding cessation was created, coded 1 (one) for those children who stopped breastfeeding after a known duration and 0 (zero) for those who continued to breastfeed up to the end of the study period (age 24 months for SWS and 15 months for ALSPAC). Children still being breastfed at the end of the study period were considered censored cases and contributed proportionally to the median breastfeeding time.

The estimate of breastfeeding median and 95% confidence interval were estimated using the Kaplan Mayer nonparametric estimator. The log‐rank test was applied to verify the equality of survival distributions between the subgroups of categorical variables, also using Kaplan Mayer nonparametric estimator. In the next step, the risk of breastfeeding cessation from solid food introduction was estimated using Cox's proportional hazards models to obtain crude and adjusted hazard ratios and their respective confidence intervals, both unadjusted and adjusted for confounding variables. In multivariable analyses, all available confounders were included, as they are all well‐known predictors of both breastfeeding duration and SF. The proportionality of hazard assumption between the categories of all independent variables was assessed by checking the ‐ln (−ln [survival]) versus ln (survival time) graphic, derived from survival curves. The variables that remained parallel over time were assumed to be time independent and retained in the final Cox model.

### 2.3 ETHICAL APPROVAL

The ALSPAC study was granted ethical approval by the Local Research Ethics Committee and ALSPAC Ethics and Law Committee. The SWS was approved by the Southampton and South West Hampshire Local Research Ethics Committee. The IFS 2010 was approved by the Ethics, Department of Health Sciences at the University of York.

## 3 RESULTS

After applying the inclusion criteria, there were 10,407 infants with breastfeeding duration of 2 months or more: 6,079 from ALSPAC, 1,500 from SWS, and 2,828 from IFS; 59%, 52%, and 56% of these were breastfed to the age of 6 months or beyond in ALSPAC, SWS, and IFS, respectively (Table 1). The age of the first SF varied markedly between the cohorts with 68% of the ALSPAC sample starting before 4 months and only 20% and 21%% in the SWS and IFS (Table 1). The socioeconomic characteristics of the mothers are also shown in Table S1.

In all three cohorts, there were univariate associations between breastfeeding duration and age at introduction of solids, as well as with social class, maternal age, and educational level (Table 2). Using Poisson regression, the risk of stopping breastfeeding before 6 months was highest in those starting solids before 4 months and lowest in those deferring until the age of 5 months and beyond, with little effect of adjustment for maternal sociodemographic characteristics (Table 3).

**Table 3:** Unadjusted and mutually adjusted prevalence ratio for stopping breastfeed before the age of 6 months at different ages of solid food introduction

Predictor	ALSPAC		SWS		IFS 2010	
	Crude PR (95% CI)	Adjusted PR^d^ (95% CI)	Crude PR (95% CI)	Adjusted PR^d^ (95% CI)	Crude PR (95% CI)	Adjusted PR^d^ (95% CI)
Solid food introduction						
<4 months	2.18 (1.7, 2.8)	1.90 (1.5, 2.4)	1.19 (1.0, 1.4)	1.16 (1.0, 1.4)	1.16 (1.0, 1.3)	1.14 (1.0, 1.3)
4–5 months	1.45 (1.1, 1.9)	1.37 (1.1, 1.8)	1.05 (0.9, 1.2)	1.04 (0.9, 1.2)	1.05 (0.9, 1.2)	1.05 (0.9, 1.2)
≥5 months	1	1	1	1	1	1
Mother's age						
<25 years	1.91 (1.7, 2.2)	1.42 (1.2, 1.6)	1.51 (1.2, 1.9)	1.24 (1.0, 1.6)	1.06 (0.9, 1.3)	0.95 (0.8, 1.1)
25 to 34 years	1.36 (1.2, 1.5)	1.21 (1.1, 1.4)	1.09 (0.9, 1.3)	1.07 (0.9, 1.2)	1.00 (0.9, 1.1)	0.97 (0.9, 1.1)
>34 years	1	1	1	1	1	1
Mother's qualification						
CSE/vocational^a^	2.20 (1.9, 2.5)	1.84 (1.6, 2.1)	1.58 (1.3, 1.9)	1.56 (1.3, 1.9)	‐	‐
O Level^a^	2.10 (1.9, 2.3)	1.73 (1.5, 2.0)	1.57 (1.4, 1.8)	1.52 (1.3, 1.8)		
A Level^b^	1.58 (1.4, 1.8)	1.41 (1.2, 1.6)	1.33 (1.2, 1.5)	1.31 (1.3, 1.5)		
Degree/HND	1	1	1	1		
Mother's social class^c^	2.01 (1.6, 2.5)	1.17 (0.9, 1.5)	1.54 (1.1, 2.1)	1.13 (0.8, 1.6)	1.24 (1.0, 1.5)	1.23 (1.0, 1.5)
Partly skilled/unskilled	2.03 (1.6, 2.5)	1.20 (0.9, 1.5)	1.57 (1.1, 2.2)	1.15 (0.8, 1.6)	1.19 (1.1, 1.3)	1.18 (1.1, 1.3)
Manually skilled	2.19 (1.8, 2.7)	1.34 (1.1, 1.6)	1.62 (1.2, 2.2)	1.22 (0.9, 1.7)	1.08 (1.0, 1.2)	1.07 (1.0, 1.2)
Nonmanually skilled	1.66 (1.4, 2.0)	1.27 (1.0, 1.5)	1.38 (1.0, 1.8)	1.23 (0.9, 1.7)		
Managerial/technical/professional	1	1	1	1	1	

*Note.* IFS 2010, ALSPAC, and SWS.

a Completed by the age of 16.

b Completed at the age of 18.

c IFS 2010 classification: managerial/professional, intermediate occupational, routine/manual occupational, and never worked.

d All the variables shown included in the adjusted model.

Abbreviations: ALSPAC, Avon Longitudinal Study of Parents and Children; SWS, Southampton Womans Survey; IFS 2010, Infant Feeding Survey 2010; CSE, Certificate of Secondary Education; HND, Higher National Diploma.

**Figure 1:**
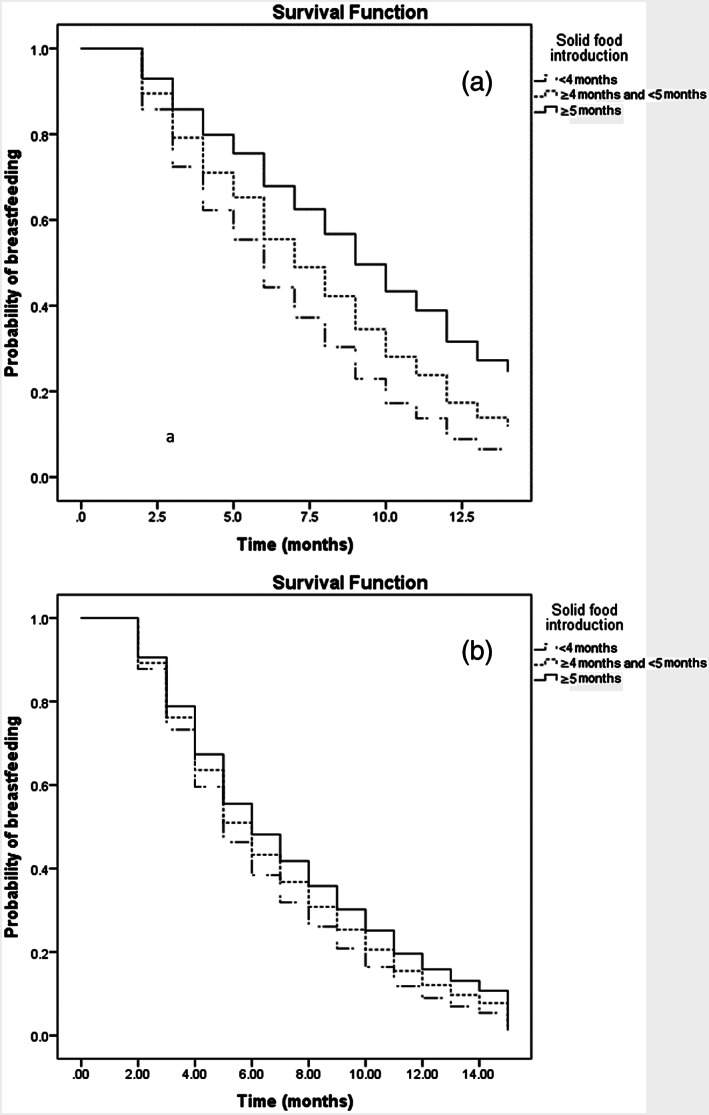
Cumulative probability of being breastfed according to the age of solid food introduction, Avon longitudinal study of parents and children (a) and Southampton Womans survey (b)

For the ALSPAC and SWS datasets, Kaplan Mayer survival curves demonstrated differences in breastfeeding duration between the three subgroups of solid food introduction (log‐rank test *p* = .001 and *p* < .001 respectively; Figure 1). The assumption of proportional hazards was not violated. Using Cox's proportional hazards modelling for ALSPAC and SWS, the hazard ratios for breastfeeding cessation remained higher for children who started solid food before or during the 4 months, with little effect of adjustment for potential sociodemographic confounders (Table 4).

**Table 4:** Crude and adjusted hazard ratio and confidence interval for breastfeeding cessation, in ALSPAC and SWS cohorts

Predictor	ALSPAC		SWS	
Solid food introduction	HR^a^ (95% CI)	HR^b^ (95% CI)	HR^a^ (95% CI)	HR^b^ (95% CI)
<4 months	2.10 (1.8, 2.4)	2.07 (1.8, 2.4)	1.32 (1.1, 1.5)	1.25 (1.1, 1.5)
4–5 months	1.52 (1.3, 1.7)	1.51 **(**1.3, 1.8)	1.15 (1.0, 1.3)	1.15 (1.0, 1.3)
≥5 months	1	1	1	1
Mother's age				
<25 years	1.85 (1.7, 2.1)	1.50 (1.3, 1.7)	1.9 (1.5, 2.6)	1.60 (1.2, 2.1)
25 to 34 years	1.38 (1.2, 1.5)	1.25 (1.1, 1.4)	1.2 (1.0, 1.4)	1.17 (1.1, 1.4)
>34 years	1	1	1	1
Mother's qualification				
CSE/vocational	1.46 (1.3, 1.6)	1.3 (1.2, 1.5)	1.43 (1.6, 1.7)	1.53 (1.2, 1.9)
O level	1.47 (1.4, 1.6)	1.3 (1.1, 1.4)	1.32 (1.1, 1.5)	1.45 (1.2, 1.7)
A level	1.22 (1.1, 1.3)	1.1 (1.0, 1.3)	1.25 (1.1, 1.4)	1.34 (1.2, 1.5)
Degree	1	1	1	1
Mother's social class				
Partly/unskilled	1.33 (1.2, 1.5)	1.0 (0.9, 1.1)	1.09 (0.9, 1.4)	0.9 (0.6, 1)
Manually skilled	1.34 (1.1, 1.6)	1.1 (0.9, 1.3)	1.13 (0.9, 1.4)	0.9 (0.7, 1.3)
Nonmanually skilled	1.48 (1.3, 1.6)	0.98 (0.8, 1.2)	1.20 (0.9, 1.6)	0.9 (0.7, 1.1)
Manager/technical	1.19 (1.1, 1.3)	0.97 (0.8, 1.1)	1.15 (0.9, 1.5)	1.0 (0.8, 1.2)
Professional	1	1	1	1

a Unadjusted.

b Mutually adjusted for all variables.

Abbreviations: ALSPAC, Avon Longitudinal Study of Parents and Children; SWS, Southampton Womans Survey; CI, confidence interval; CSE, Certificate of Secondary Education; HND, Higher National Diploma; HR, hazard ratio.

## 4 DISCUSSION

This analysis demonstrates a consistent association between the age of first SF and duration of breastfeeding in three different U.K. cohorts, studied across the period when the recommended age of the first SF in the United Kingdom increased from 4 to 6 to 6 months. It has long been recognised that breastfed infants are generally introduced to solids later (Wright, Parkinson, & Drewett, 2004), and it could easily be assumed that this association reflects confounding, as breastfeeding mothers are generally more affluent and better educated and may well show different adherence to feeding recommendations. However, in our analyses, restricted only to mothers who breastfed for at least 2 months with adjustment for education and occupational class, a dose–response association was still found in all three cohorts. A limitation is that we could not adjust for other possible confounders, such as general health‐seeking behaviour, but as these are themselves likely to show some social gradient, the absence of attenuation after adjustment for sociodemographic characteristics suggests that these may not have greatly modified the effect.

Early breastfeeding cessation has been described in the literature as a result of many factors, including health care, socioeconomic, biological, psychological, and cultural factors (Cohen et al., 2018; Oakley, Henderson, Redshaw, & Quigley, 2014; Ruowei Li & Chen, 2008). Two recent systematic reviews and a meta‐analysis (Cohen et al., 2018; Mangrio, Persson, & Bramhagen, 2018) examined various factors associated with breastfeeding initiation and duration but only focused on sociodemographic factors. Earlier studies have described an association between early solid food introduction and cessation of breastfeeding (Noble & Emmett, 2006; Simard et al., 2005). Noble and Emmett found reduced milk intake associated with solid food introduction in a subsample of breastfed ALSPAC children with food intake recorded, but this was a cross‐sectional analysis, so total breastfeeding duration was not considered. This study thus set out to examine this association in large pre‐existing cohorts, which already held the relevant data, taking a prospective approach and exploring the extent to which this association can be explained by sociodemographic factors.

This study had limitations inherent in a secondary analysis of data, which were not collected expressly for this purpose, or for between cohort comparison. The different surveys reported sociodemographic factors differently, and SF and breastfeeding information was not collected at exactly the same age or with exactly the same wording. For example, in the SWS, the age for SF was reported in retrospect, and the modal reported age was exactly 4 months. It seems likely that some of these may actually have started SF just before 4 months, but we had to treat this as 4–5 months for this analysis. We were also not able to use survival analysis for all three studies, but we could apply Poisson analysis to all three.

A strength of our analysis is that although the three studies used different tools and approaches, they used similar, well‐standardised methodologies for the collection of the variables required and between them provided over 10,000 breastfeeding infants. This makes this analysis well powered to detect relatively small effect sizes, which would nonetheless have important public health implications.

The three cohorts did differ in their sampling methodologies. ALSPAC and SWS were each restricted to one geographical area of the United Kingdom, with a high proportion of eligible mothers recruited (Boyd et al., 2013; Inskip et al., 2006). In contrast, IFS was sampled from throughout the United Kingdom, including regions with much lower rates of breastfeeding. Furthermore, they oversampled the most deprived mothers in order to achieve reasonable numbers of responses from all social strata (McAndrew et al., 2012). However, only around one sixth of the original sample had sufficient data to be included in this analysis, and in their report, they stated that they found a lower response rate in areas of higher deprivation and among younger mothers, so the sample was not fully representative of the United Kingdom (McAndrew et al., 2012). Despite this, the three cohorts had remarkable similar initial breastfeeding rates. IFS had the largest fall off in breastfeeding between birth and our age of inclusion. This was probably because in this analysis, the eligible age for inclusion for IFS participants was, by necessity, at 10 weeks, rather than the 8‐week cut‐off used in the other cohorts, as this is a period when breastfeeding rates are falling rapidly in the United Kingdom.

The three cohorts represented very different SF eras. In the earliest (ALSPAC), two thirds of children started solids before the age of 4 months, at time when official advice was to start from 4 months. Since 2003, mothers have been advised to wait till 6 months and never to start before 4 months, and by the time of the last cohort, less than a quarter did so. Generally, the strongest effects were seen in the earliest cohort, which was both the largest and had the highest proportion starting solids very early. The strongest association in all cohorts was seen with SF started before 4 months but even starting between 4 and 5 months was associated with an increased risk of breastfeeding cessation before 6 months in ALSPAC and IFS, whereas in both ALSPAC and SWS, SF starting between 4 and 5 months was also associated with an overall shorter breastfeeding duration. This dose–response relationship suggests a potentially causal association, and the minimal attenuation resulting from adjustment for sociodemographic factors makes residual confounding by incompletely measured socioeconomic factors unlikely.

A putative mechanism for this is the displacement of milk production by solid food, leading to secondary lactation failure. Infant suckling is one of the most important stimuli to milk production and in its absence, or reduced frequency, milk production is expected to decline. An early observational study found that suckling and breast milk consumption were reduced when solid foods were offered (Drewett, Phil, Payman, & Whiteley, 1987), and a randomised controlled trial (Bajaj, Dubey, Nagpal, Singh, & Sachdev, 2005) showed an inverse relationship between the energy density of semi‐solid foods and energy intake from breast milk. More recently, a trial that compared commencement of solids at 4 versus 6 months in exclusively breastfed infants and measured breast milk intake using stable isotopes found that those starting solids earlier consumed 10% less breast milk than those still exclusively breastfeeding (Wells et al., 2012). These studies demonstrate that infants self‐regulate their energy consumption, resulting in breast milk displacement by semi‐solid foods. What is not clear is whether this displacement would be sufficient to lead to secondary lactation failure in some instances. A large Swedish cohort study observed reduced breastfeeding frequency after commencement of solids, but no association between time of solid food introduction and duration of breastfeeding (Hornell et al., 2001). However, only a small proportion of Hornell's participants introduced solids without also introducing formula milk, so the study would have been underpowered to detect an effect.

Hornell's study found that most breastfeeding mothers introduced some formula and that this was associated with a much steeper decline in breastfeeding frequency and reduced breastfeeding duration (Hornell et al., 2001). Thus, an alternative explanation is that starting solids is associated with also starting formula milk and that it was actually the introduction of formula milk at the same time as starting solids that led to earlier cessation. We were not able to test this hypothesis, as the cohorts did not have consistent measures of formula intake over time. Even if this is the true underlying mechanism, it would still suggest that deferring the recommended age of first SF would also defer introduction of formula milk. It is also possible that mothers were introducing solids because they were facing challenges with breastfeeding and felt that they did not have enough breastmilk. Only controlled trials can truly test whether the relationship between the age of first solids and breastfeeding duration is causal, and such trials are very rare. However, a recent trial that randomised parents to either conventional complementary feeding or a “baby‐led approach,” where solids were introduced significantly later, did find that breastfeeding duration was 4 weeks longer in the baby‐led group (Taylor et al., 2017).

These three surveys illustrate the effect of different recommendations for solid food introduction on actual practice. ALSPAC was conducted well before and SWS mainly before 2003, when the recommendation for starting SF was still specified as “from 4 to 6 months,” and in these surveys, a majority of breastfeeding mothers actually started SF before 4 months. In contrast the IFS, conducted 7 years after the change in recommendation to delay SF until 6 months, showed a much later average age of first solids. In the IFS report, the authors were able to compare the age of SF in a series of their surveys before and after the change in recommendation in 2003 and showed a marked increase in age of first solids immediately after that date (McAndrew et al., 2012). Whereas much else may have changed in the intervening years, this rapid change demonstrated the effect of the consistent public health message that was adopted in the United Kingdom after 2003 (McAndrew et al., 2012). However, whereas nearly half of mothers in IFS delayed until beyond 5 months, very few did so beyond 6 months. If all mothers most commonly introduce solids in the month prior to the then recommended age, any relaxation in the recommended age is likely to result in much earlier SF. This is important not only in terms of breastfeeding duration but also because early solid food introduction has also been independently associated with other health risks (Wright et al., 2004).

In conclusion, evidence from the three large surveys consistently demonstrates that early introduction of SF predicts a shorter breastfeeding duration and suggests that deferring SF is important to sustain breastfeeding. This confirms the importance for public health of maintaining consistent messages to parents that SF should be delayed until 6 months and the importance of continued breastfeeding after solids have started (SACN, 2018).

## CONFLICTS OF INTEREST

The authors declare that they have no conflicts of interest.

## CONTRIBUTIONS

AL undertook all the analyses and produced the first draft. PE, SR, and KG worked on the design and analysis of their cohorts' infant data. CW, AG, and AL planned the analysis, and PE, SR, and SC advised on the data available, enabled us to access it, and commented on the paper in draft. CW created the final draft, and all the authors have read and approved it. This publication is the work of the authors and will serve as guarantors for the contents of this paper.

## Supporting information

Table S1 Questions in each original questionnaire for building information about breastfeeding duration and solid food introduction
Table.S2 Variables available on the dataset and its categorisation in the Survival Analysis
Table.S3 Variables available on the dataset and its categorisation in the Poisson Regression
