# Supplementary material for: Does early introduction of solid feeding lead to early cessation of breastfeeding?
Source: Matern Child Nutr. 2020 Jan 29;16(4):e12944. doi: 10.1111/mcn.12944 (PMC7507438; doi:10.1111/mcn.12944)
Supplement: Supplementary file 1 — Table S1 Questions in each original questionnaire for building information about breastfeeding duration and solid food introduction Table.S2 Variables available on the dataset and its categorisation in the Survival Analysis Table.S3 Variables available on the dataset and its categorisation in the Poisson Regression [file MCN-16-e12944-s001.doc]

**Supplementary information - Appendix**

**Table 1 - Questions in each original questionnaire for building** information about breastfeeding duration and solid food introduction

|  | Breastfeeding duration | Solid food introduction |
| --- | --- | --- |
| **IFS-2010** | **Section 2: About the milk that you give your baby** | **Section 4: About other drinks and food that you may give to your baby** |
|  | Q8. Has your baby EVER been given breast milk (via syringe, bottle or cup etc.) or have you put your baby to the breast, even if this was only once?  Q9. How old was your baby when he/she was LAST given breast milk or you put them to your breast? Please write the age in the appropriate box Either in days  OR In whole weeks plus any additional days: | Section 4: About other drinks and food that you may give to your baby Q36. Has your baby ever had any foods such as cereal, rusks, baby rice, fruit, vegetables or any other kind of solid food? Q37. How old was your baby when he/she first had any food apart from milk? Please write a number in the box Please write in the age to the nearest whole week Q38. At present, are you regularly giving your baby cereal, rusks, baby rice or any other solid food? |
| **SWS** | **6 MONTH INFANCY QUESTIONNAIRE** | **6 MONTH INFANCY QUESTIONNAIRE** |
|  | 1 MILK OR FORMULA FEEDING  1.1 Did you ever put your baby to the breast, even for a single feed? 1.4 Are you still breast feeding? 1.5 How old was your baby when he/she last had a breast feed? or On what date did he/she last have a breast feed? | 4 INTRODUCTION OF FOODS AND SUPPLEMENT USE 4.1 How old was he/she when solids were first regularly introduced? or On what date were solids first regularly introduced? |
|  | **12 MONTH INFANCY**  **QUESTIONNAIRE** | **12 MONTH INFANCY**  **QUESTIONNAIRE** |
|  | 1 MILK OR FORMULA FEEDING   - 1. Was he/she breast fed after the age of 6 months?   2. Are you still breast feeding?   1.4 How old was your baby when he/she last had a breast feed? or On what date did he/she last have a breast feed? | 3 INTRODUCTION OF FOODS  3.1 Was he/she eating solid foods by 6 months of age?  3.2 How old was he/she when solids were first regularly introduced?  or On what date were solids first regularly introduced? |
|  | **24 MONTH CHILD**  **QUESTIONNAIRE** |  |
|  | 1. MILK OR FORMULA FEEDING    1. Are you still breast feeding?   16.3 How old was your baby when he/she last had a breast feed? or  On what date did he/she last have a breast feed? | - |
|  | **3 YEAR CHILD**  **QUESTIONNAIRE** |  |
|  | 1. FOOD FREQUENCY  1.2 * Which types of milk has your child used regularly in drinks and added to breakfast cereals over the past 3 months? *(list up to 3 below)* | - |

|  | **Breastfeeding duration** | **Solid food introduction** |
| --- | --- | --- |
| ALSPAC | **MY YOUNG BABY - 4 week** |  |
|  | SECTION B:FEEDING  B1. How have you fed your baby since she was born? Please indicate for each of the times given.   1. First 24 hours b) Rest of 1st week c) 2nd week d) 3rd week e) 4th week   B4. a) How is your baby being fed at the moment? breast 1 Bottle 2 breast and bottle 3 other 4 | - |
|  | **MY DAUGHTER/SON - 6 months** | **MY DAUGHTER/SON - 6 months** |
|  | SECTION C:FEEDING  C1. Has your baby ever had the following:  Age started   1. bottle of ordinary b) powdered follow-on c) soya milk d) goat's milk e) hypo-allergenic f) ordinary cow's milk C2. Did you breast feed?   Yes, I am still breast feeding Yes, I breast fed 2 How old was the baby months weeks but have now stopped when you stopped? I never breast fed | SECTION C:FEEDING  C3.a) In how many meals a day does she eat solids now? |
|  | **MY INFANT SON/DAUGHTER - 15 months** | **MY INFANT SON/DAUGHTER - 15 months** |
|  | SECTION D:FEEDING  D2. Was he/she breast fed?  Yes, he/she is still being breast fed  Yes, was breast fed but now stopped  How old was he/she when months breastfeeding stopped?  He/She was never breast fed | D20. Babies first solid meals are usually a puree. When did your child first start having meals with lumps in? Age started (months) |

Table 2 – Variables available on the dataset and its categorization in the Survival Analysis

| **Dataset** | **Variable** | **Label** | **Categorization** |
| --- | --- | --- | --- |
| **ALSPAC** |  | | |
| **Outcome** | Time_kc403a | Time in months - ALSPAC | In months, continuous from 2mo |
|  | Status_kc403a | Status | Censored (0)  Event (1) |
| **Predictors** | kbagesolid_teste | Solid food introduction | <4mo (0)  ≥4mo and <5mo (1)  ≥5mo (2) |
|  | Mother_Age_mz028b | Mother's age | <25y (0)  25 to 34y (1)  ≥35y (2) |
|  | Mother_Qualification_c645a | Mother's qualification | CSE-Vocational (0)  O level (1)  A level (2)  Degree (3) |
|  | Social_Class_755 | Mother’s social class | V unskilled- IV partly skilled (1)  III skilled manual (2)  III skilled no-manual (3)  II management-technical (4)  I Professional (5) |
| **SWS** |  | | |
| **Outcome** | Time_agelbfm | Time in months - SWS | In months, continuous from 2mo |
|  | Status_agelbfm | Status | Censored (0)  Event (1) |
|  | agelbfm15mo | Time in months 1 | In months, continuous from 2mo.  To be equal time as ALSPAC. All children who had BF beyond the 15mo were coded as censored. |
| **Predictors** | Solid_introduction_agesld | Solid food introduction | <4mo (0)  ≥4mo and <5mo (1)  ≥5mo (2) |
|  | jwage1 | Mother's age | <25y (1)  25 to 34y (2)  ≥35y (3) |
|  | awexam1 | Mother’s qualification | CSE-none (1)  O level (2)  A level (3)  HND-Degree (4) |
|  | Social_Class_awsc | Mother’s social class | IV partly skilled-V unskilled (1)  III skilled manual (2)  III skilled no-manual (3)  II management/technical (4)  I professional (5) |

Table 3- Variables available on the dataset and its categorization in the Poisson Regression

| **Dataset** | **Variable** | **Label** | | **Categorization** |
| --- | --- | --- | --- | --- |
| **ALSPAC** |  | | | |
| **Outcome** | kc403a1 | | BF prevalence at 6mo | Yes (0)  No (1) |
|  | Kc403x | | Breastfeeding duration | In months |
|  | BF_3_categories | | BF 3 categories (excluded BF<2mo) | <4mo (1)  ≥4mo and ≤6mo (2)  >6mo (3) |
| **Predictors** | kbagesolidx | | Solid food introduction x | ≥5mo (0)  ≥4mo and <5mo (1)  <4mo (2) |
|  | mz028_x1 | | Mother's age x1 | ≥35y (0)  25 to 34y (1)  ≤24y (2) |
|  | c645b_Mother_qualification | | Mother's qualification | Degree (0)  A level (1)  O level (2)  Vocational-CSE (3) |
|  | c755b_social_class | | Mother's social class | I Professional (0)  II management-technical (1)  III skilled no-manual (2)  III skilled manual (3)  IV partly skilled/unskilled (4) |
| **SWS** |  | | | |
| **Outcome** | agelbfm1 | | BF prevalence at 6m | Yes (0)  No (1) |
|  | agelbfm | | Age last breast fed, completed months | In months |
|  | BF_3_categories | | BF 3 categories (excluded BF<2mo) | <4mo (1)  ≥4mo and ≤6mo (2)  >6mo (3) |
| **Predictors** | agesldx | | Solid food introduction x | ≥5mo (0)  ≥4mo and <5mo (1)  <4mo (2) |
|  | jwagex | | Mother’s age x | ≥35y (0)  25 to 34y (1)  ≤24y (2) |
|  | awexam1 | | Mother's qualification | Degree/HDN (0)  A level (1)  O level (2)  CSE-none (3) |
|  | awsc1 | | Mother's social class | I professional (0)  II management/technical (1)  III skilled no-manual (2)  III skilled manual (3)  IV partly skilled/ unskilled (4) |
| **IFS-2010** |  | | | |
| **Outcome** | Q61 | BF prevalence 6mo | | Yes (0)  No (1) |
|  | Q6 | Q6. How old was your baby when he/she was LAST given breast milk or you put them to your breast? | | 8 categories (from up to 10w to more than 9mo)  The category 9 (not stated) was converted in missing |
|  | BF_3_categories | BF 3 categories (excluded BF<2mo) | | <4mo (1)  ≥4mo and ≤6mo (2)  >6mo (3) |
| **Predictors** | Q363 | Solid Food Introduction new | | ≥5mo (0)  ≥4mo and <5mo (1)  <4mo (2) |
|  | mage21 | Mothers age 1 | | ≥35years (0)  25 to 34years (1)  ≤24years (2) |
|  | nssec31 | Mother’s social class1 | | Managerial-professional (0)  intermediate occupational (1) routine/manual occupation (2)  never worked (3) |
